# Suppression of the Oncogenic Transcription Factor FOXM1 by Proteasome Inhibitors

**DOI:** 10.1155/2014/596528

**Published:** 2014-06-24

**Authors:** Andrei L. Gartel

**Affiliations:** Department of Medicine, University of Illinois at Chicago, Chicago, IL 606012, USA

## Abstract

The oncogenic transcription factor FOXM1 is one of the key regulators of tumorigenesis. We found that FOXM1 upregulates its own transcription and its protein stability depends on its interaction with the chaperone nucleophosmin. We also determined that FOXM1 is negatively regulated by the tumor suppressor p53. We identified the thiazole antibiotics Siomycin A and thiostrepton as inhibitors of transcriptional activity and FOXM1 expression via proteasome inhibition. In addition, we found that all tested proteasome inhibitors target FOXM1. We showed synergy between thiostrepton and bortezomib in different human cancer cell lines and *in vivo.* We generated isogenic human cancer cell lines of different origin with wild-type p53 or p53 knockdown and we demonstrated that proteasome inhibitors induce p53-independent apoptosis in these cells. Using RNA-interference or proteasome inhibitors to inhibit FOXM1 we found that suppression of FOXM1 sensitized human cancer cells to apoptosis induced by DNA-damaging agents or oxidative stress. We encapsulated thiostrepton into micelle-nanoparticles and after injection we detected accumulation of nanoparticles in tumors and in the livers of treated mice. This treatment led to inhibition of human xenograft tumor growth in nude mice. Our data indicate that targeting FOXM1 increases apoptosis and inhibits tumor growth.

## 1. Introduction

Forkhead box M1 (FOXM1) is a member of the Forkhead family of transcription factors that regulate expression of genes involved in cell cycle progression and maintenance of genomic stability [1]. It has been shown that FOXM1 is overexpressed in a variety of human solid tumors (reviewed in [2–4]) in diffuse large B-cell lymphoma [5], while its expression is suppressed in nondividing cells [1]. In addition, FOXM1 induces metastasis and epithelial-mesenchymal-like transition in hepatocellular carcinoma (HCC) [6]. On the other hand, suppression of FOXM1 delayed liver tumor growth in mice [7, 8] and inhibited the metastatic potential of human cancer cells* in vitro* [9]. Recently, we pointed out [4] that FOXM1 contributes to all hallmarks of cancer described by Hanahan and Weinberg in [10]. Particularly FOXM1 sustains proliferation, evades action of tumor suppressors, increases resistance of cancer cells to apoptosis, induces replicative immortality, stimulates angiogenesis, contributes to invasion and metastasis, and enables genomic instability and inflammation [4]. These data suggest that FOXM1 is a potent oncogenic transcription factor that could be a bona fide target in cancer [11]. In this paper, I describe studies on the regulation and targeting of FOXM1 that were performed in my laboratory in recent years.

## 2. Positive and Negative Regulation of FOXM1 in Cancer

When we compared levels of FOXM1 in normal human fibroblasts, fibroblasts with p53 knockdown, and corresponding immortal/oncogenic cell lines with inactivated p53, we found that partial deletion or inactivation of p53 leads to upregulation of FOXM1 expression [12]. Similarly, p53 knockdown in several human cancer cell lines with wild-type p53 led to upregulation of FOXM1 mRNA and protein expression, while induction of p53 by DNA damage led to downregulation of FOXM1. Our data imply that p53 inhibits FOXM1 transcription. Inactivation of p53 may lead to upregulation of FOXM1 in human cancer cells. In addition, we showed that radiation and DNA-damaging agents may increase FOXM1 levels in human tumors with p53 mutations [12]. Our data [12] and results from another group [13] suggest that one of the functions of tumor suppressor p53 is to control expression of the FOXM1 oncogene.

We found that FOXM1 binds and activates its own proximal promoter (unpublished data) and is involved in a positive autoregulatory loop [14] that is partially responsible for activation of its expression. Since FOXM1 transcriptional inhibitors also inhibit FOXM1 protein expression [15], FOXM1 autoactivation is required for proper FOXM1 expression. It is not clear whether a positive feedback loop exists for other members of the Forkhead family or only for FOXM1. Additional experiments are needed to better understand the biological significance of the FOXM1 autoregulatory loop.

Using mass spectrometric analysis and immunoprecipitation/Western blot experiments, we showed that the chaperone nucleophosmin (NPM) forms a complex with FOXM1 [16]. Several functionally different cellular proteins interact with NPM, implying that NPM is implicated in numerous processes in the cell. Colocalization of FOXM1 and NPM was confirmed by immunofluorescence microscopy. Furthermore, knockdown of NPM in human cancer cells led to suppression of FOXM1, indicating that NPM controls levels of FOXM1 in cancer cells. In addition, we found that FOXM1, which is usually in the nucleus, colocalized with NPM in the cytoplasm in the OCI/AML3 leukemia cell line with mutant cytoplasmic NPM. These data suggested that NPM is able to regulate the intracellular localization of FOXM1. Overall NPM interacts with FOXM1 and their interaction is critical for expression and cellular localization of FOXM1 in cancer cells. Therefore, NPM is required for oncogenic activity of FOXM1. We propose that inhibiting the interactions between FOXM1 and NPM by chemical compounds or small specific peptides will suppress FOXM1 expression in cancer cells.

## 3. Thiazole Antibiotics/Proteasome Inhibitors Target FOXM1

We suggested that FOXM1 might be “the Achilles' heel of cancer” [17] and an attractive target for cancer treatment. Using a high-throughput, cell-based assay system, we isolated the thiazole antibiotic Siomycin A as an inhibitor of FOXM1 [18]. Next, we found that the structurally similar thiazole antibiotic thiostrepton also inhibits the transcriptional activity and FOXM1 expression, and they both induce strong apoptosis in human cancer cells of different origin that correlates with suppression of FOXM1 [19]. Specifically, we showed that neuroblastoma, leukemia, liver cancer [19], prostate cancer [20], and melanoma cells [21] are sensitive to low micromolar concentrations of Siomycin A and thiostrepton. Furthermore, we found that both thiazole antibiotics specifically inhibited FOXM1 activity and expression but did not inhibit activity or expression of other members of the Forkhead family.

We demonstrated that the anticancer activities of thiostrepton and Siomycin A are linked to their activity as proteasome inhibitors in human tumor cells [22]. We tested whether other known thiazole antibiotics such as berninamycin, micrococcin P1 and micrococcin P2, thiocillin, and YM-266183 (lacking the quinaldic acid ring B) [23] could demonstrate proteasome inhibitor activity. These thiazole antibiotics could not act as proteasome inhibitors and could not inhibit growth of human cancer cells. Moreover, structural modification of thiostrepton to thiostrepton methyl ester (with an open inactive B ring) also did not demonstrate this activity. These data suggest that the B ring of thiostrepton and Siomycin A, which is absent in other thiazole antibiotics, together with ring A that is present in all thiazole antibiotics determines the proteasome inhibitory activity and anticancer properties of these drugs [23].

We also found that all tested proteasome inhibitors such as bortezomib, MG132, and others inhibit FOXM1 transcriptional activity and FOXM1 expression, suggesting that negative regulation of FOXM1 by proteasome inhibitors is a general feature of these drugs that contributes to their anticancer properties [22]. Since all proteasome inhibitors inhibit the transcriptional activity of FOXM1 and FOXM1 expression, we proposed the following model of FOXM1 suppression by proteasome inhibitors [24]: all proteasome inhibitors increase expression of a putative negative regulator of FOXM1 (NRFM) that binds FOXM1 to inhibit transcriptional activity of FOXM1 on the FOXM1 promoter because of the FOXM1 autoregulation mechanism. As a consequence of inhibition of FOXM1 transcriptional activity on its own promoter, expression of FOXM1 mRNA and protein diminishes after treatment with proteasome inhibitors [22]. This hypothesis may explain why all tested proteasome inhibitors block FOXM1 transcriptional activity and inhibit expression independently of their structures.

NAC (N-acetyl-L-cysteine) is used to inhibit ROS activity and to test the activity of ROS (reactive oxygen species) inducers. However, we identified NAC as a novel inhibitor of proteasome inhibitors that was able to prevent cellular effects linked to proteasome inhibition, such as suppression of FOXM1, stabilization of multiple proteins, apoptosis, and accumulation of ubiquitin conjugates [25]. Most importantly, we demonstrated using NMR that NAC, but not other known ROS scavengers, binds to proteasome inhibitors to inhibit their activity. It appears that NAC is the first compound that may antagonize the activity of proteasome inhibitors [25].

Previously, we showed that proteasome inhibitors suppress FOXM1 and induce apoptosis in human cancer cell lines of different origin. We decided to examine the role of p53 in proteasome inhibitor-induced apoptosis using isogenic human colon, breast, and liver cancer cell lines that differ only in their p53 status, and we found that the proteasome inhibitors induce p53-independent apoptosis [20]. Apoptosis stimulated by proteasome inhibitors was linked to p53-independent induction of the proapoptotic protein Noxa. Proteasome inhibitors also suppressed growth of several human cancer cell lines independently of p53 status. In addition, thiostrepton induced more robust apoptosis in HepG2 liver cancer cells with p53 knockdown than in parental cells with wild-type p53, suggesting that in some cases p53 may protect against proteasome inhibitor-induced cell death [20]. Our data generated in isogenic human cancer cell lines with different p53 status suggest that proteasome inhibitors largely induce p53-independent apoptosis in human cancer cells.

Combination of anticancer drugs is a viable strategy for the treatment of cancer. Using combinations of drugs at nontoxic concentrations may reduce the probability of cancer cells becoming drug-resistant. We tested the combination of the thiazole antibiotic/proteasome inhibitor thiostrepton and the proteasome inhibitor bortezomib (Velcade) in various human tumor cell lines [20, 26]. We found that sublethal concentrations of these two drugs stimulated potent apoptosis and led to inhibition of long-term colony formation. The synergistic relationship between thiostrepton and bortezomib in different human cancer cell lines was confirmed by calculating their combination index that was as low as 0.2 [26]. This combination index suggested that the interaction of these drugs is highly synergistic. Their synergy was explained by their proteasome inhibitory activities that differently target the proteasome, because thiostrepton modification that did not have proteasome inhibitory activity did not show any synergy in combination with bortezomib [25].

## 4. Suppression of FOXM1 Sensitizes Cancer Cells to Apoptosis Induced by Anticancer Drugs

The role of the transcription factor FOXM1 in apoptosis is not understood well. We decided to examine functions of FOXM1 in apoptosis by developing human cancer cell lines with stable FOXM1 knockdown and analyzing their responses to different anticancer agents. First, we treated MIA PaCa-2 (pancreatic cancer) and MDA-MB-231 (breast cancer) vector controls and FOXM1 knockdown (KD) human cell lines with increasing concentrations of the DNA-damaging agent, doxorubicin. We showed that the FOXM1-KD cell lines were more sensitive to programmed cell death induced by doxorubicin [27]. Next, we tested if transient knockdown of FOXM1 by small interfering RNAs (siRNA) would result in increased cell death after treatment with doxorubicin. We determined that cells with transient FOXM1-KD also were more sensitive to apoptosis after DNA damage [27]. MIA PaCa-2 and MDA-MB-231 vector controls and FOXM1-KD cells were also treated by ionizing radiation and the extent of apoptosis was determined by flow cytometry. We showed that FOXM1-KD cells were much more sensitive to apoptosis induced by ionizing radiation [27]. Since FOXM1 is suppressed by proteasome inhibitors, we decided to test combination treatment of human cancer cells with proteasome inhibitors together with DNA-damaging agents. Human cancer cells were treated with the combination of doxorubicin/*γ*-irradiation and bortezomib/thiostrepton and they underwent stronger cell death compared to treatments with single agents. We tested the effect of combinatorial treatment on long-term survival by performing clonogenic assay. Human cancer cells were treated with *γ*-irradiation along with thiostrepton/bortezomib and the colony forming ability was assessed 10 days later. Combinatorial treatment drastically inhibited colony formation compared to control cells [27]. Using additional experiments we demonstrated that FOXM1 may inhibit apoptosis via suppression of proapoptotic JNK and activation of antiapoptotic Bcl-2 [27]. These data suggest that suppressing FOXM1 in human cancer cells could make them more sensitive to DNA-damaging agents. Since proteasome inhibitor bortezomib is already in clinic, it can be used with current DNA-damaging chemotherapeutic agents for treatment of cancer patients.

Proteasome inhibitors suppress FOXM1, but paradoxically knockdown of FOXM1 additionally sensitizes human cancer cells to apoptosis induced by proteasome inhibitors [28]. Control vector and shFOXM1 human cancer cell lines were treated with thiostrepton, MG132, and bortezomib for 24 h. Using caspase-3 cleavage as a readout we found that knockdown of FOXM1 increased cell death in human cancer cells induced by proteasome inhibitors about 2-fold. These data suggest that suppressing FOXM1 in human cancer cells could make them more sensitive to proteasome inhibitors [28].

It has been shown that FOXM1, which is overexpressed in a majority of human tumors, protects cancer cells from oxidative stress. We proposed that the combination of FOXM1 suppression and induction of ROS by specific drugs would specifically kill human cancer cells. We demonstrated that FOXM1 suppression by RNAi elevates intracellular ROS levels and overexpression of the scavenger MnSOD that is target of FOXM1 partially reversed this effect [29]. These data verified the notion that FOXM1 exerts its antioxidant activity partially through the activation of MnSOD. Furthermore, we demonstrated that human cancer cell lines with FOXM1-KD became more sensitive to ROS-induced cell death than original cell lines [29]. Since RNA interference currently is not a feasible treatment option, we tested proteasome inhibitors as FOXM1 inhibitors in combination with ROS inducers* in vitro* and* in vivo*. We found that the combinations of ROS inducers with FOXM1/proteasome inhibitors stimulated robust apoptosis in human cancer cell lines. The proteasome inhibitor bortezomib in combination with the ROS inducer *β*-phenylethyl isothiocyanate suppressed the growth of human breast tumor xenografts in nude mice [29]. Since bortezomib is already in the clinic and the ROS inducer PEITC is in clinical trials, our data suggest that induction of ROS and inhibition of FOXM1 by these drugs may be used for cancer patients. This combinatorial treatment will have fewer side effects and will be less toxic, because normal cells generally express very low levels of FOXM1 and ROS [29].

## 5. Targeting FOXM1 in Mouse Models

We demonstrated earlier that Siomycin A and thiostrepton suppressed tumor growth in a human breast cancer xenograft model [30]. However, we decided to improve the delivery of thiazole antibiotics and use nanoparticles for this purpose. Since thiostrepton is highly insoluble, we encapsulated it into micelles assembled from amphiphilic lipid-PEG within the inner lipid compartment of the micelle [31]. Developed micelle-thiostrepton nanoparticles were found to be about 100 nm in diameter and −16 mV in zeta potential [31]. The benefits of utilizing nanoparticles for delivering anticancer drugs are linked to the increase of the concentration of drug delivered into the tumor and to the protection of the drug from external stimuli in circulation during delivery [31, 32]. After continuous treatment with nanoparticle-encapsulated thiostrepton, we observed decreased xenograft tumor growth induced by the human MDA-MB-231 breast and HepG2 liver cancer cell lines. We detected accumulation of nanoparticles in xenograft tumors and in the livers of treated mice. Maximum accumulation of thiostrepton into xenograft tumors was observed 4 hours after administration. In addition, we showed that* in vivo* suppression of oncogenic FOXM1 by the proteasome inhibitor thiostrepton correlated with increased apoptosis in drug-treated tumors [31]. The strong anticancer effect of micelle-thiostrepton nanoparticles* in vivo* and their accumulation in the liver suggest that this nanoparticle-thiostrepton formulation may be potentially useful for treatment of liver cancer [31].

We showed proapoptotic synergy between bortezomib and thiostrepton in human cancer cell lines [26]. It has been suggested that inhibition of the “caspase-like” site of the proteasome by thiostrepton can sensitize inhibition of the “chymotrypsin-like” and “trypsin-like” sites of the proteasome by bortezomib [33]. We decided to test this combination in MDA-MB-231 xenograft breast tumors* in vivo* [32]. To determine the anticancer effect of bortezomib and thiostrepton, animals with MDA-MB-231-luc breast xenograft tumors were treated with either bortezomib (0.5 mg/kg) or micelle-encapsulated thiostrepton (30 mg/kg) or a combination of both [32]. Xenograft tumors stably expressed luciferase and bioluminescence data correlated with the tumor growth curve. Using both methods we showed that xenograft tumors treated with a combination of bortezomib and thiostrepton were much smaller in size compared to those treated with single drugs or with nontreated tumors [32]. Using different apoptosis markers, we showed that the suppression of tumor growth by bortezomib/thiostrepton is associated with induction of cell death within the tumor cells. We showed before [31] that thiostrepton-micelles have affinity for accumulation into the liver. Therefore, we decided to test the anticancer activity of thiostrepton-micelles in combination with bortezomib in the mouse liver cancer model [32]. Induction of hepatocellular carcinoma (HCC) in mice was attained by intraperitoneal (IP) injection of the carcinogen diethylnitrosamine (DEN) into 2-week-old neonatal male mice simultaneously with phenobarbital (PB; 0.67 mg/mL) that was administered in the drinking water. 32 weeks following the DEN/PB protocol, animals were separated into 4 groups of (1) nontreated control, (2) thiostrepton-micelle only 30 mg/kg, IV, (3) bortezomib only 0.5 mg/kg, IP, and (4) thiostrepton-micelle 30 mg/kg and bortezomib 0.5 mg/kg [32]. Animals were treated 15 times before being sacrificed, after which livers were analyzed for tumors. The majority of hepatocellular tumors in all treatment groups were adenomas with maximum number in nontreated animals and minimum adenomas in animals after combination treatment [32]. The body weight of treated animals did not decrease significantly compared with control animals, indicating that the toxicities of individual or combinatorial treatments were low. Our data imply that combinatorial treatment of solid tumors with thiostrepton and bortezomib may have a therapeutic potential [32].

We decided to examine the possibility of suppressing FOXM1 in xenograft tumors* in vivo* through the administration of FOXM1-specific siRNA [34]. We used a polyethylimine-based cationic polymer, JetPEI (Polyplus) as an encapsulation agent for the* in vivo* delivery agent for FOXM1-siRNA by direct intratumoral injection at a dose of 10 *μ*g siRNA/tumor, 3 times per week [34]. First, we demonstrated that PEI-encapsulated siRNA could be retained in xenograft tumors after intratumoral injection for 24 hours [34]. After 10 injections tumors were resected and analyzed for protein and mRNA levels of FOXM1 and its transcriptional targets. We found that the protein levels of FOXM1 and FOXM1 transcriptional targets, Aurora B Kinase, and cdc2B were diminished in tumors treated with FOXM1-siRNA [34]. Suppression of FOXM1, Aurora B Kinase, and cdc25B corresponded to an increase in protein levels of CDK inhibitor p27 that is indirectly negatively regulated by FOXM1. These data suggest that the direct injection of PEI-encapsulated FOXM1-siRNA into xenograft tumor leads to functional FOXM1 suppression. Overall, our data suggest that targeting FOXM1 in tumors with FOXM1 inhibitors individually or in combination with other anticancer drugs may have strong therapeutic potential against cancer.
